# Editorial: Crosstalk between the Osteogenic and Neurogenic Stem Cell Niches: How Far are They from Each Other?

**DOI:** 10.3389/fncel.2015.00504

**Published:** 2016-01-19

**Authors:** Wanda Lattanzi, Maria Concetta Geloso

**Affiliations:** ^1^Institute of Anatomy and Cell Biology, Faculty of Medicine and Surgery “A. Gemelli”, Università Cattolica del Sacro CuoreRome, Italy; ^2^Latium Musculoskeletal Tissue BankRome, Italy

**Keywords:** stem cell niche, osteogenic niche, neural stem cells (NSCs), mesenchymal stem cells (MSCs), regeneration

Despite the intense research on adult neural stem cell biology suggested possible translational outcomes in regenerative medicine for neurodegenerative diseases, neuroregeneration is unlikely to occur in adult brain, due to intrinsic features that characterize the neural stem cell niche.

Mesenchymal stem cells (MSCs), osteogenic stem cells residing in the bone marrow stroma (also named bone marrow stromal cells), have been long considered highly plastic multipotent precursors, able to commit toward diversified lineages, including non-mesodermal ones. Their *in vitro* plasticity and ease of processing prompted their wide, sometimes untimely, exploitation in diversified regenerative medicine applications (Park et al., 2012; Bianco et al., 2013). They have been tested also for their putative, yet widely debated, neuroregenerative potential. This controversial issue stimulated this Research Topic, which aims to delve into relevant scientific milestones addressing the differences, possible interconnections, and overlaps between the osteogenic and the neurogenic niches' biology.

The debated neuronal transdifferentiation potential of MSCs recently led to their inappropriate exploitation for the treatment of neurodegenerative disorders. The regulatory and ethical issues regarding this topic have been discussed in the Opinion paper by Solarino et al., delving into a recent Italian case of medical malpractice, which triggered significant international dispute (Abbott, 2013; Blasimme and Rial-Sebbag, 2013). Indeed, a better clarification of the specific features displayed by the osteogenic and the neurogenic stem cell niches is needed, as discussed by Lattanzi et al. This mini-review provides a pairwise comparison of the two niches within their *in vivo* environments, highlighting functionally relevant similarities and differences that should be considered to achieve a more rational clinical translation.

The contribution by Salgado et al. provides an exhaustive description of osteogenic and neural stem cells' features, focusing on their possible interaction within the brain environment. In particular, the MSCs' secretome is known to exert autocrine and paracrine effects that may be relevant for potential therapeutic exploitations, also in the central nervous system (Ribeiro et al., 2011; Drago et al., 2013; Kim et al., 2013; Sart et al., 2014; Wright et al., 2014).

The role of neural crest stem cells (NCSCs) in regulating the bone marrow niche is provided in the review by Coste et al. NCSCs are capable of epithelial-to-mesenchymal transition, and ultimately give rise to both neural precursors and nestin-positive MSCs, actively involved in the homeostatic regulation of the hematopoietic stem cell niche (Achilleos and Trainor, 2012; Mayor and Theveneau, 2013).

A significant overlap between the two niches relies on the molecular (Wnt, NOTCH, FGF, TGF-BMP, SHH signaling pathways) and secretome (BDNF, NGF, VEGF, PDGF) profiles, along with the intimate relationship with vessels, being a common structural feature observed in adult stem cell niches.

Diverse phylogenetically old signaling pathways, including nucleotides and neuropeptides, are shared between the osteogenic and the neurogenic niches, exerting trophic, and immunomodulatory functions. Cavaliere et al. exhaustively discussed the often opposing roles played by purinergic ligands. These establish a common paracrine pathway that modulates MSCs' and NSCs' activity, in both physiological and pathological conditions. They appear to be involved in the crosstalk between the two niches, by modulating the immune response, which triggers stem cell recruitment after stressful insults (Cavaliere et al.).

Among neuropeptides, the direct effects of neuropeptide Y (NPY), mediator for signaling in both neurogenic and osteogenic niches, has been reviewed by Geloso et al., with special attention to its effects on neurogenic niche. Data indicating a direct pro-neurogenic effect of NPY on NSCs, as well as the concomitant modulatory action on astrocytes, microglia, and endothelium activities within the niche have been discussed. Interestingly, a possible crosstalk between released nucleotides and NPY related pathways emerges (Jia and Hegg, 2012), suggesting that they could also represent a point of intersection between shared ancient molecular pathways.

Neurotransmitters released by the sympathetic nervous system, interestingly including NPY, as recently reviewed by Park et al. (2015), are known to be also involved in the regulation of hematopoietic stem cell (HSC) functions, mainly acting on endothelial cells and nestin-positive MSCs, which retain HSCs. In this regard, the relevance of catecholaminergic modulation of hematopoiesis has been extensively reviewed by Cosentino and coworkers (Cosentino et al.), highlighting their established role in the complex network of neural and neuroendocrine agents that regulate stem cell biology (Cosentino et al.).

Within the wide range of external stimuli acting on the epigenetic control of adult tissue stem cell niches, the effects of extremely-low frequency electromagnetic field (ELFEF) stimulation is emerging as a tool to modulate neurogenic and osteogenic processes, as discussed by Leone et al. They highlighted the possible shared pathways induced by ELFEFS on both niches, including Wnt/beta-catenin signaling and the activation of p300 or other histone acetyltransferases by Runx2 (Leone et al.).

The interdependence of brain and skull during development seems to rely also on the role of interposed meninges (Richtsmeier and Flaherty, 2013). Within this intriguing topic, Bifari et al. provided findings showing the distribution of neural precursor markers in rat meninges during development up to adulthood, related to the newly identified niche function of meninges (Decimo et al., 2011).

Finally, an interesting evolutionary perspective on the relation between osteogenesis and neurogenesis is provided in the opinion paper by Boeckx and Benítez-Burraco, who approached this topic from a different “biolinguistic” standpoint. The Authors postulated that critical genes active in the osteogenic niche (including homeogenes, e.g. DLXs, morphogens, e.g. BMPs, and the master regulatory RUNX2 gene), hence giving rise to skull globularity in anatomically modern humans, also have important consequences in brain development and plasticity, ultimately leading to our distinctive mode of cognition (Boeckx and Benítez-Burraco).

Taken together, the papers included in this research topic seem to suggest an emerging cross-domain scenario in which significant molecular signaling and biological features are shared between osteogenic and neurogenic stem cells niches. The two niches appear to be interconnected in evolution, during development, and further beyond. Nonetheless, relevant differences in the relative stem cell niche dynamics should not be neglected, in order to appropriately design potential cross-lineage tissue regenerative strategies.

## Author contributions

Both Authors contributed equally in conceiving, drafting, revising, and finalizing the present manuscript.

### Conflict of interest statement

The authors declare that the research was conducted in the absence of any commercial or financial relationships that could be construed as a potential conflict of interest.
